# Mediterranean Diet and White Blood Cell Count—A Randomized Controlled Trial

**DOI:** 10.3390/foods10061268

**Published:** 2021-06-02

**Authors:** Álvaro Hernáez, Camille Lassale, Sara Castro-Barquero, Nancy Babio, Emilio Ros, Olga Castañer, Anna Tresserra-Rimbau, Xavier Pintó, Miguel Ángel Martínez-González, Dolores Corella, Jordi Salas-Salvadó, Ángel M. Alonso-Gómez, José Lapetra, Miquel Fiol, Enrique Gómez-Gracia, Lluis Serra-Majem, Emilio Sacanella, Ana García-Arellano, José V. Sorlí, Andrés Díaz-López, Montserrat Cofán, Ramón Estruch

**Affiliations:** 1 August Pi i Sunyer Biomedical Research Institute (IDIBAPS), 08036 Barcelona, Spain; sacastro@clinic.cat (S.C.-B.); eros@clinic.cat (E.R.); esacane@clinic.cat (E.S.); mcofan@clinic.cat (M.C.); restruch@clinic.cat (R.E.); 2 Consorcio CIBER, M.P. Fisiopatología de la Obesidad y Nutrición (CIBEROBN), Instituto de Salud Carlos III, 28029 Madrid, Spain; classale@imim.es (C.L.); nancy.babio@urv.cat (N.B.); ocastaner@imim.es (O.C.); annatresserra@ub.edu (A.T.-R.); xpinto@bellvitgehospital.cat (X.P.); mamartinez@unav.es (M.Á.M.-G.); dolores.corella@uv.es (D.C.); jordi.salas@urv.cat (J.S.-S.); angelmago13@gmail.com (Á.M.A.-G.); joselapetra543@gmail.com (J.L.); miguel.fiol@ssib.es (M.F.); lserra@dcc.ulpgc.es (L.S.-M.); agarare@gmail.com (A.G.-A.); jose.sorli@uv.es (J.V.S.); andres.diaz@urv.cat (A.D.-L.); 3 Blanquerna School of Health Sciences, Universitat Ramon Llull, 08025 Barcelona, Spain; 4 Centre for Fertility and Health (CeFH), Norwegian Institute of Public Health, 0473 Oslo, Norway; 5 Cardiovascular Risk and Nutrition Research Group, Hospital del Mar Medical Research Institute (IMIM), 08003 Barcelona, Spain; 6 Department of Medicine, Faculty of Medicine and Health Sciences, University of Barcelona, 08036 Barcelona, Spain; 7 Universitat Rovira i Virgili, Departament de Bioquimica i Biotecnologia, Unitat de Nutrició Humana, 43201 Reus, Spain; 8 Institut d’Investigació Pere Virgili (IISPV), 43204 Reus, Spain; 9 Lipid Clinic, Endocrinology and Nutrition Service, Hospital Clínic, 08036 Barcelona, Spain; 10 Department of Nutrition, Food Science and Gastronomy, XaRTA, INSA, Faculty of Pharmacy and Food Sciences, University of Barcelona, 08028 Barcelona, Spain; 11 Lipids and Vascular Risk Unit, Internal Medicine Service, Hospital Universitario de Bellvitge, 08907 L’Hospitalet de Llobregat, Spain; 12 Department of Preventive Medicine and Public Health, Universidad de Navarra, 31008 Pamplona, Spain; 13 Department of Nutrition, Harvard TH Chan School of Public Health, Boston, MA 02115, USA; 14 Department of Preventive Medicine, Universidad de Valencia, 46010 Valencia, Spain; 15 Bioaraba Health Research Institute, Osakidetza Basque Health Service, Araba University Hospital, University of the Basque Country UPV/EHU, 01009 Vitoria-Gasteiz, Spain; 16 Research Unit, Department of Family Medicine, Distrito Sanitario Atención Primaria Sevilla, 41013 Sevilla, Spain; 17 Health Research Institute of the Balearic Islands (IdISBa), Hospital Son Espases, 07120 Palma de Mallorca, Spain; 18 Department of Preventive Medicine and Public Health, Universidad de Málaga, 29071 Málaga, Spain; egomezgracia@uma.es; 19 Instituto de Investigación Biomédica de Málaga (IBIMA), 29010 Málaga, Spain; 20 Instituto de Investigaciones Biomédicas y Sanitarias, Universidad de Las Palmas de Gran Canaria, 35016 Las Palmas, Spain; 21 Centro Hospitalario Universitario Insular Materno Infantil (CHUIMI), Servicio Canario de Salud, 35016 Las Palmas, Spain; 22 Internal Medicine Service, Hospital Clínic, 08036 Barcelona, Spain; 23 Servicio Navarro de Salud (Osasunbidea), 31003 Pamplona, Spain; 24 Serra Hunter Fellow, Universitat Rovira i Virgili, 43201 Reus, Spain; 25 Nutrition and Mental Health Research Group (NUTRISAM), Universitat Rovira i Virgili, 43201 Reus, Spain

**Keywords:** white blood cell count, Mediterranean diet, leukopenia, leukocytosis, randomized controlled trial, prevention

## Abstract

We aimed to assess the effects of the antioxidant-rich Mediterranean diet (MedDiet) on white blood cell count. Our study population included participants in the PREvención con DIeta MEDiterránea study (average age 67 years old, 58% women, high cardiovascular risk). We assessed whether a MedDiet intervention enriched in extra-virgin olive oil or nuts, versus a low-fat control diet, modified the incidence of leukocytosis (>11 × 10^9^ leukocytes/L), mild leukopenia (<4.5 × 10^9^ leukocytes/L), or severe leukopenia (<3.5 × 10^9^ leukocytes/L) in individuals without the condition at baseline (*n* = 3190, *n* = 2925, and *n* = 3190, respectively). We also examined whether MedDiet modified the association between leukocyte count alterations and all-cause mortality. Both MedDiet interventions were associated with a lower risk of developing leukopenia (incidence rates: 5.06% in control diet, 3.29% in MedDiet groups combined; hazard ratio [95% confidence interval]: 0.54 [0.36–0.80]) and severe leukopenia (incidence rates: 1.26% in control diet, 0.46% in MedDiet groups combined; hazard ratio: 0.25 [0.10–0.60]). High cumulative adherence to a MedDiet was linked to lower risk of leukocytosis (incidence rates: 2.08% in quartile 1, 0.65% in quartile 4; HR_Q4-Q1_: 0.29 [0.085–0.99]) and attenuated the association between leukopenia and all-cause mortality (*P*-interaction = 0.032). In brief, MedDiet decreased the incidence of white blood cell count-related alterations in high cardiovascular risk individuals.

## 1. Introduction

There is growing evidence that diet can modulate the response of the immune system [1,2]. Experimental and clinical research in nutritional immunology has uncovered several food components, such as dietary antioxidants (vitamin E, some phenolic compounds), omega-3 polyunsaturated fatty acids, folates, vitamin A, zinc, and probiotics as modifiable factors capable of impacting immune function [3,4,5,6,7,8]. However, immunomodulatory effects of some of these nutrients are yet to be proven beyond low-grade inflammation (particularly in the case of vitamin E and phenolic antioxidants) [3,4,9,10]. Combining these functionally diverse bioactive compounds in a healthy dietary pattern such as the Mediterranean diet (MedDiet) may be a sound strategy to promote the overall function of the immune system. In observational studies, adherence to the MedDiet has been consistently associated with lower rates of cardiovascular disease [11], cancer [12], and all-cause mortality [13]. There is also first-level evidence from the large-scale randomized clinical trial PREDIMED (PREvención con DIeta MEDiterránea) on the efficacy of the MedDiet to reduce incident cardiovascular disease [14], type 2 diabetes [15], and certain types of cancer [16] in older individuals at high cardiovascular risk. Adherence to a MedDiet has also been associated with improvements in other immune-related responses such as cytokine patterns [17,18], gut-derived immunity [19], and thrombosis [20,21]. A better functioning of the immune system could be a mediator of these beneficial effects [22,23,24,25]. However, no intervention study has assessed the effects of MedDiet on other immune-related responses such as white blood cell (WBC) count. High and low WBC count have both been associated with increased mortality [26,27,28,29] and are related to inadequate responses of the immune system. On the one hand, high counts are intimately linked to chronic low-grade inflammation [30] and the incidence of inflammation-related disease in the general population and in individuals at high cardiovascular risk [31]. On the other hand, low WBC levels are associated with nutritional deficiencies, chronic use of certain medications, and different states of immune dysfunction such as autoimmune diseases, cancers of the immune system, infections, and diseases of bone marrow, spleen, and blood [32,33]. WBC counts have been shown to be potentially modulated by changes in the nutritional status in cross-sectional or short-term studies [32,33]. Thus, an improvement in WBC counts and their related conditions after following a MedDiet is plausible.

Our primary aim was to assess the association of MedDiet with WBC count in middle-aged and older individuals at high cardiovascular risk participating in the PREDIMED study. Our secondary aim was to determine whether following a MedDiet modulated the association between WBC count alterations and all-cause mortality.

## 2. Materials and Methods

### 2.1. Study Population

The study population were participants in the PREDIMED trial. It was a multicenter, randomized, controlled trial conducted in Spain assessing the effects of the MedDiet intervention on the primary prevention of cardiovascular outcomes in individuals at high cardiovascular risk. Particularly, three intervention arms were compared: (1) a MedDiet enriched with extra-virgin olive oil (MedDiet–EVOO), (2) a MedDiet enriched with mixed nuts (MedDiet–Nuts), and (3) a low-fat control diet. Eligible participants were men (aged 55–80 years) and women (aged 60–80 years) with no previous history of severe cardiovascular disease at baseline, but presenting type 2 diabetes or three or more of the following risk factors: smoking, hypertension, low-density lipoprotein cholesterol ≥160 mg/dL, high-density lipoprotein cholesterol ≤40 mg/dL, overweight/obesity, and family history of premature coronary heart disease [14,34]. Enrollment began on 25 June 2003, and the last participant was recruited on 30 June 2009. The PREDIMED study was registered under the International Standard Randomized Controlled Trial Number ISRCTN35739639. The study protocol complied with the Declaration of Helsinki and was approved by the institutional review boards of all recruiting centers. An institutional ethics committee (CEIC-PSMAR) approved the particular protocol of this sub-project (code: 2018/8180/I, date: 4 December 2018). The study protocol, recruiting methods, and data collection processes have been described elsewhere [14,34]. All volunteers provided written informed consent before joining the trial.

**Figure 1:**
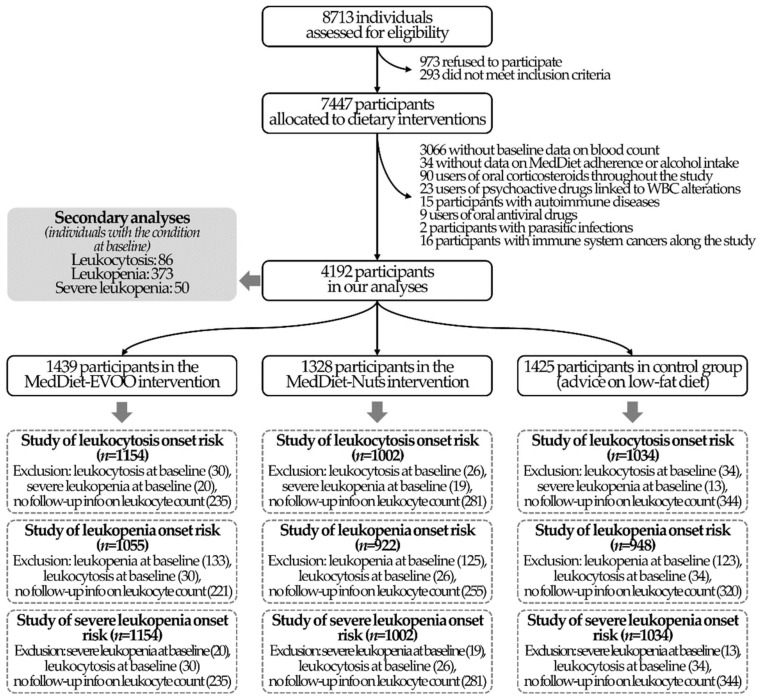
Study flowchart.

The aim of this sub-study is to investigate the effects of MedDiet on WBC count, which was not a predetermined endpoint in the PREDIMED study protocol. Of the 4381 participants recruited in the PREDIMED centers where complete blood count was collected in yearly visits, we excluded 34 participants without baseline data on MedDiet adherence or ethanol intake. To exclusively ascertain the effect of the dietary intervention on WBC levels, we excluded individuals with any potential condition linked to WBC count alterations [32,33], such as: (1) users of medications associated with alterations in WBC count at any point of the study (90 users of oral corticosteroids and 23 users of psychoactive drugs linked to WBC count alterations, such as bupropion, clozapine, lamotrigine, lithium, and valproate); (2) participants who presented an autoimmune disease (15 individuals, determined as the use of immunosuppressant medications in any study follow-up); (3) individuals who reported any viral infection (nine participants, determined as the use of oral antiviral medication in any study follow-up) or parasitism (two participants, determined as the use of oral anti-parasitic medication in any study follow-up); and (4) participants who developed any cancer of the immune system throughout the study (16 individuals). No individuals with health outcomes related to alterations in WBC count (severe blood cell and bone marrow conditions, such as aplastic anemia, overactive spleen, and myelodysplastic syndromes; congenital immune diseases, such as myelokathexis and Kostmann syndrome; sarcoidosis; or having undergone a splenectomy) were included in the trial. This yielded a main analytical sample of 4192 individuals. In the analyses related to the incidence of WBC count alterations, we also excluded individuals with leukocytosis or leukopenia at baseline or without information on WBC count in the follow-up visits. The flowchart of the study is available in Figure 1.

### 2.2. Dietary Intervention

Volunteers were randomly assigned to one of the three intervention arms on a 1:1:1 ratio. MedDiet interventions promoted (1) the consumption of plant-based foods (such as fruits, vegetables, legumes, and nuts) and fish; (2) the use of extra-virgin olive oil as a main culinary fat; (3) a decrease in the intake of sugary drinks, commercial bakery goods, sweets, pastries, and fat spreads; (4) the substitution of red/processed meats for poultry; and (5) the consumption of foods prepared by home-made methods (such as the traditional “sofrito”, a stir-fried sauce of tomato, garlic, onion, or leeks sautéed in olive oil). To boost compliance and account for family needs, volunteers allocated in the MedDiet–EVOO intervention received 1 L/week of extra-virgin olive oil and those in the MedDiet–Nuts group were provided with 210 g/week of mixed nuts plus three monthly 1 kg packs. Volunteers allocated to the low-fat control group were advised (1) to promote the intake of fruits, vegetables, legumes, low-fat dairy products, lean fish, and seafood; and (2) to decrease their consumption of vegetable oils (including olive oil), commercial bakery goods and sweets, nuts and fried snacks, red and processed fatty meats, visible fat in meats and soups, fatty fish, seafood canned in oil, spread fats, and sofrito. Further details of the dietary protocol have been described [14,34,35].

As a secondary measurement of dietary exposure, we also estimated the adherence of study participants to a MedDiet at each visit using the MedDiet adherence score. It was a validated short screener questioning whether the volunteer followed 14 essential dietary traits related to a MedDiet. It scored positively on the consumption of (1) olive oil (used as main fat for cooking and seasoning earned 1 point, an intake of ≥4 tablespoons/day earned an additional 1 point); (2) vegetables (≥2 servings/day); (3) fruit (≥3 servings/day); (4) mixed nuts (≥3 servings/week); (5) legumes (≥3 servings/week); (6) fish/seafood (≥3 servings/week); (7) poultry and rabbit over red/processed meat (1 point, plus an additional 1 point if the consumption of red/processed meat was <1 serving/day); (8) <1 serving per day of butter, margarine, or cream; (9) <1 carbonated/sugar-sweetened beverage per day; (10) <2 servings per week of non-homemade sweets/pastries; (11) wine in moderation (100 mL/day, within meals); and (12) a sofrito-based dish at least twice per week [36].

### 2.3. Outcomes

First, WBC count was measured in fasting plasma samples in an automated analyzer as previously described [37]. The normal range for WBC count in male and non-pregnant female adults is usually defined as 4.5 to 11.0 × 10^9^ cells/L [38]. Thus, leukopenia was defined as presenting ≤4.5 × 10^9^ cells/L, and leukocytosis as ≥11 × 10^9^ cells/L. Considering that other studies have used stricter definitions in the particular case of leukopenia [39], we additionally defined “severe leukopenia” as presenting ≤3.5 × 10^9^ cells/L. We calculated incidence and time-to-event of the onset of any of these three conditions among volunteers without extreme WBC count at the beginning of the study. We defined “onset” as the appearance of any of the above conditions in one of the follow-ups that lasted until the last visit for which data is available. We considered as a valid incident case any presentation of a WBC count alteration that persisted for at least three subsequent follow-up visits and had no more than one “return to normal” value.

For our secondary analysis, we collected information on all-cause mortality. A clinical event committee checked any fatal outcome up to 1 December 2010, as well as the date of death, using the information available from follow-up study visits, yearly review of medical records between 2011 and 2017, repeated contact with the participants, and the national death registry [14,34].

### 2.4. Covariates

Trained personnel collected data on age; sex; educational level; prevalence of diabetes, hypercholesterolemia, hypertriglyceridemia, and hypertension; body mass index; and smoking habits [14,34]. We extracted the hemoglobin values (a key covariate in WBC-related analyses [40]) from the same complete blood count from which the WBC count came [37]. We estimated physical activity levels in metabolic equivalents of task-minute per day from the Minnesota Leisure-Time Physical Activity Questionnaire validated for the Spanish adult population [41,42]. Finally, we estimated alcohol intake (in g/day) from a semi-quantitative 137-item food frequency questionnaire, also validated in Spanish adults [43].

### 2.5. Power Analysis

The number of individuals and cases in each study group allowed us to detect as significant (*p*-value <0.05, ≥80% power) hazard ratios (HR) for the comparisons between the low-fat control diet and (1) both MedDiet interventions combined, (2) MedDiet–EVOO, and (3) MedDiet–Nuts, respectively, of the following values: ≤0.43, 0.33, and 0.30 in relation to leukocytosis onset; ≤0.59, 0.54, and 0.50 regarding leukopenia onset; and ≤0.32, 0.24, and 0.17 regarding severe LEUKOPENIA (Table S1). We performed these analyses using the “powerSurvEpi” package in R Software (Vienna, Austria) [44].

### 2.6. Statistical Analyses

We described baseline characteristics of the volunteers using means and standard deviation (normally distributed continuous variables), medians and interquartile range (non-normally distributed continuous variables), and proportions (categorical variables). We compared our study population with the subset of non-included volunteers of the PREDIMED study by one-way analysis of variance tests for normally distributed continuous variables, Kruskal–Wallis tests for non-normally distributed continuous variables, and Pearson chi-squared tests for categorical variables.

We used three sets of Cox proportional hazards regression models where the outcomes were the risk of developing leukocytosis, leukopenia, and severe leukopenia. Follow-up time was defined as the time between the date of enrollment and (1) the midpoint between the last visit without the outcome and the first visit in which the WBC abnormality was registered [45]; (2) 6 years of maximum follow-up time; or (3) 1 December 2010, whichever came first. We investigated whether there were differences in the risk of presenting the outcome in the two MedDiet intervention groups (combined and individually) relative to the control diet. We adjusted Cox models for sex (strata variable), recruitment site (strata variable), educational level (primary, secondary, tertiary, unavailable information; strata variable), WBC count at baseline (continuous), age (continuous), diabetes (yes/no), hypercholesterolemia (yes/no), hypertriglyceridemia (yes/no), hypertension (yes/no), smoking habit (current/former/never smoker), hemoglobin levels (continuous), leisure-time physical activity (continuous), body mass index (continuous), alcohol consumption (continuous), and two propensity scores that used 30 baseline variables to estimate the probability of assignment to each of the intervention groups. We used robust variance estimators to account for intra-cluster correlations [14] and fitted models using the “survival” package in R Software [46]. We also depicted incident cases using Kaplan–Meier cumulative incidence curves for study groups using inverse probability weighting with a propensity score model of assignment to intervention or control group based on the covariates above listed.

We investigated the effects of MedDiet interventions on 6-year WBC count evolution as a continuous variable by repeated measurement mixed models. We assessed time effects (continuous change per year in the whole study population) and between-group changes (difference in changes over time in MedDiet intervention groups, combined and individual, relative to the control diet). Models included the same covariates as above and were fitted using the “lme4” package in R Software [47].

In addition to the intention-to-treat approach, we considered adherence to the MedDiet as exposure. We calculated the cumulative average MedDiet adherence as the mean of all the 14-point MedDiet adherence score values until the occurrence of the event (incident cases) or the last study visit with available data (non-cases). We used this variable as continuous and in quartiles. Cox models were stratified and adjusted for the same covariates as in the intention-to-treat analyses plus the PREDIMED intervention group. In addition, we used multiple linear regressions with cubic spline models to analyze the dose-response association between the cumulative adherence to a MedDiet and the cumulative mean of WBC count throughout the study (calculated similarly as for MedDiet adherence score). We set the reference cut-point at the minimum observed value (5 points out of 14). Cumulative means of WBC count whose values departed from the average by more than two standard deviations were considered outliers and excluded from the analyses. We performed the previous analyses in the whole study population and stratifying volunteers in quartiles according to WBC count at baseline (after testing whether there was a significant interaction between baseline WBC count and the cumulative MedDiet adherence, by applying a likelihood ratio test between the nested models with the interaction product-term and without it). Models included the same covariates as for survival analyses according to cumulative MedDiet adherence. Splines were fitted and plotted using the “gam” package in R Software [48].

Finally, we determined whether MedDiet modified the association between WBC count alterations at baseline and all-cause mortality. We compared the volunteers: (1) allocated to the MedDiet interventions relative to those in the control group; and (2) with a high cumulative MedDiet adherence (above the median) relative to those with low values (below the median). To determine whether the interaction between MedDiet group and WBC alterations was significant, we fitted Cox models where the outcome was all-cause mortality and applied a likelihood ratio test between the nested models with and without an interaction product-term of “WBC count alteration × group”.

A two-sided *p*-value < 0.05 was considered significant. We performed all analyses with R Software, version 3.5.0 (Vienna, Austria) [49].

## 3. Results

### 3.1. Study Population

Our study sub-sample were elderly adults (mean age 67 years, 58% women) with high prevalence of cardiovascular risk factors at baseline (84% hypertension, 74% hypercholesterolemia, 49% obesity, 46% diabetes, 30% hypertriglyceridemia, 14% current smokers) (Table 1). Individuals in the analytical sample were less likely to have type-2 diabetes and more prone to present hypercholesterolemia, hypertriglyceridemia, hypertension, and obesity than the PREDIMED participants not included in the analyses (Table S2). The numbers of individuals considered in the analyses on the onset of leukocytosis, leukopenia, and severe leukopenia were 3190, 2925, and 3190, respectively. Median follow-up time was 3.2 years.

### 3.2. Intervention with MedDiet and Incidence of WBC Count Alterations

As seen in Table 2, both MedDiet interventions combined were associated with 46% less risk of developing leukopenia (HR = 0.54 [95% confidence interval: 0.36; 0.80]). The association was significant and of a similar magnitude when individual MedDiet intervention groups were considered (HR_MedDiet–EVOO_ = 0.59 [0.38; 0.93], HR_MedDiet–Nuts_ = 0.47 [0.29; 0.78]). In addition, MedDiet interventions were also linked to lower incidence of severe leukopenia (HR_MedDiets combined_ = 0.25 [0.10; 0.60], HR_MedDiet–EVOO_ = 0.31 [0.11; 0.85], HR_MedDiet–Nuts_ = 0.18 [0.055; 0.60]). No significant effects were found for leukocytosis. Weighted Kaplan–Meier curves are available in Figure S1.

Despite the effect of MedDiet interventions on the risk of leukopenia, WBC count increased throughout follow-ups in the whole study population (+0.026 × 10^9^ cells/L·year [0.003; 0.049]) but this trend was not different between study groups (Table S3).

### 3.3. MedDiet Adherence and WBC Count

**Figure 2:**
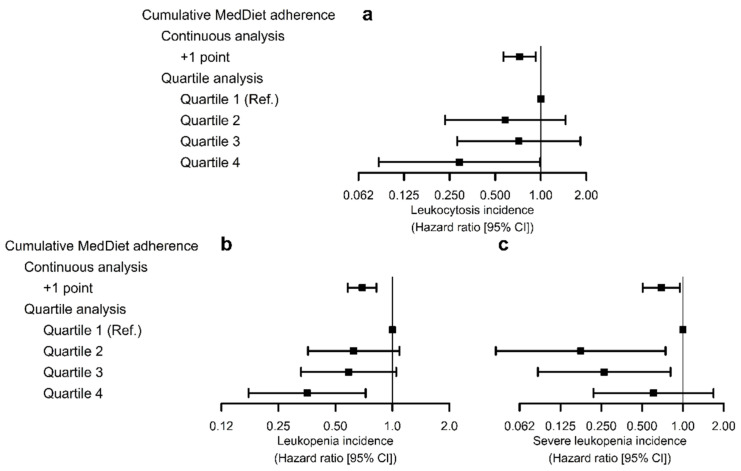
Associations of cumulative MedDiet adherence and risk of leukocytosis (**a**), leukopenia (**b**), and severe leukopenia (**c**). Hazard ratios were estimated by multivariable Cox regression models stratified by sex, recruitment site, and educational level, and adjusted for white blood cell count, age, diabetes, hypercholesterolemia, hypertriglyceridemia, hypertension, smoking habit, leisure-time physical activity, body mass index, hemoglobin levels, alcohol consumption (baseline values, all), and PREDIMED intervention group. We used robust standard errors to account for intra-cluster correlations.

High cumulative MedDiet adherence was associated with lower risk of developing leukocytosis (+1 point in cumulative adherence: HR = 0.72 [0.57; 0.92]), mainly due to individuals with greater adherence (HR_Q4 vs. Q1_ = 0.29 [0.085; 0.99]; Figure 2a). We also observed a linear association between increasing MedDiet adherence and lower risk of leukopenia (+1 point in cumulative adherence: HR = 0.69 [0.58; 0.82]; HR_Q4 vs. Q1_ = 0.35 [0.17; 0.72]; Figure 2b). Finally, we detected an association between cumulative MedDiet adherence and reduced risk of severe leukopenia, possibly non-linear due to the limited amount of incident cases of the outcome (+1 point in cumulative adherence: HR = 0.69 [0.51; 0.95]; HR_Q2 vs. Q1_ = 0.18 [0.042; 0.74], HR_Q3 vs. Q1_ = 0.26 [0.085; 0.81], HR_Q4 vs. Q1_ = 0.61 [0.22; 1.68]; Figure 2c).

**Figure 3:**
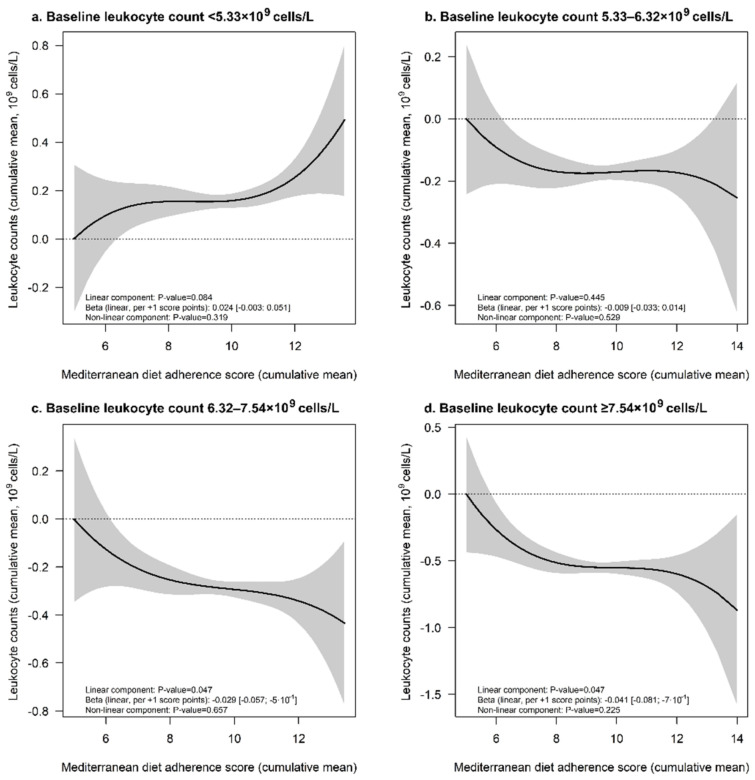
Associations between cumulative means of MedDiet adherence scores and white blood cell count throughout the study stratified according to baseline levels: first (**a**), second (**b**), third (**c**), and fourth quartiles (**d**). Dose-dependent associations between cumulative means of MedDiet adherence scores and cumulative means of white blood cells were estimated by multiple linear regressions with cubic spline models. Models were adjusted for white blood cell count at baseline, age, sex, recruitment site, educational level, diabetes, hypercholesterolemia, hypertriglyceridemia, hypertension, smoking habit, leisure-time physical activity, body mass index, hemoglobin levels, alcohol consumption (baseline values, all), and PREDIMED intervention group.

Cumulative MedDiet adherence was unrelated to the cumulative mean of WBC count (−0.011 × 10^9^ cells/L [−0.027; 0.004], *p*-value for linear association = 0.14; *p*-value for non-linear association = 0.11). However, associations between these two parameters were different according to baseline WBC count (*p*-value for the interaction between baseline count and cumulative MedDiet adherence < 0.001). A positive relationship between cumulative MedDiet adherence and WBC count was suggested in volunteers with low baseline levels (first quartile); on the contrary, inverse associations were found in the third and fourth quartile of baseline WBC count (Figure 3).

### 3.4. Interaction between WBC Count-Related Alterations and MedDiet on All-Cause Mortality

**Figure 4:**
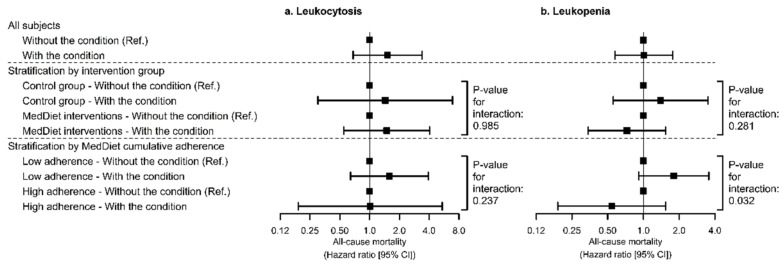
Associations of leukocytosis (**a**) and leukopenia (**b**) with all-cause mortality risk stratified by MedDiet groups. Hazard ratios were estimated by multivariable Cox proportional hazards regression models stratified by sex, recruitment site, and educational level, and adjusted for white blood cell count, age, diabetes, hypercholesterolemia, hypertriglyceridemia, hypertension, smoking habit, leisure-time physical activity, body mass index, hemoglobin levels, and alcohol consumption (at baseline). Analyses stratified according to cumulative MedDiet adherence were further adjusted for PREDIMED intervention group. We used robust standard errors to account for intra-cluster correlations.

Leukocytosis was not associated with all-cause mortality, modulated or not by MedDiet (Figure 4a). However, high adherence to a MedDiet attenuated the relationship between leukopenia and all-cause mortality (HR_below-median adherence_ = 1.80 [0.92; 3.55], HR_above-median adherence_ = 0.54 [0.19; 1.54], *p*-interaction = 0.032; Figure 4b). Exact values of HRs are available in Table S4.

## 4. Discussion

Our results show that a MedDiet intervention was associated with a lower risk of developing mild and severe leukopenia in middle-aged and older adults at high cardiovascular risk. In addition, high adherence to a MedDiet—regardless of the intervention—was associated with decreased incidence of any WBC count alteration and attenuated all-cause mortality risk linked to leukopenia.

Our main finding is the protective effect of MedDiet on onset of leukopenia (both MedDiet interventions combined decreased the risk by 46%), reducing, in particular, the incidence of severe leukopenias (by 75%). In addition, high adherence to the MedDiet attenuated mortality risk in individuals with low WBC count. Leukopenia is a well-known indicator of frailty, particularly when one is suffering an infectious disease. Its presence has been shown to be highly prevalent in hospitalized patients with confirmed COVID-19 infection [50], is associated with increased severity of respiratory disease by Klebsiella pneumoniae [28], Staphylococcus aureus [29], and Mycobacterium tuberculosis [51], and is a predictor of mortality in patients with yellow fever [27] and after acute care surgery [52]. Low WBC counts are known to appear in nutritional deficits, chronic use of certain medications, and clinical outcomes such as autoimmune diseases, cancers of the immune system, infections, and bone marrow, blood, and spleen disorders [32,33]. Thus, the association of the MedDiet with a lower incidence of leukopenia and the attenuation of the leukopenia-related mortality risk suggests a beneficial impact of this dietary pattern in immune function due to the improvement in the nutritional status after the MedDiet interventions [32,33].

High adherence to the MedDiet was additionally related to a decreased risk of developing leukocytosis. Previous cross-sectional analyses [53,54] and a short-term, small-scale intervention study with a MedDiet–inspired dietary pattern [55] have reported similar results. Adherence to the Dietary Approaches to Stop Hypertension eating plan and lower scores in the Dietary Inflammatory Index (increments in diet quality) were associated with reduced WBC levels [56,57], and a short-term, small-scale intervention based on a vegan diet (plant-rich as the MedDiet) has also been shown to decrease WBC count [58]. High WBC count is a marker of low-grade inflammation [30,59] and has been associated with a greater incidence of inflammation-related disease in the general population and in individuals at high cardiovascular risk [31]. Thus, the anti-inflammatory capacity of the MedDiet (which has already been associated with decreased levels of pro-inflammatory cytokines such as C-reactive protein, interleukin-6, tumor necrosis factor α, macrophage chemotactic protein-1, and soluble intercellular adhesion molecule-1 in the PREDIMED study [17,18]) may contribute to explaining this beneficial effect on WBC count. Several MedDiet bioactive compounds act synergistically to promote this anti-inflammatory effect. First, antioxidants (present in extra-virgin olive oil, nuts, fruits, vegetables, and legumes) neutralize reactive species of oxygen and nitrogen. This, in turn, decreases the excessive activation of nuclear factor kappa beta, the main cellular regulator of inflammatory responses, through the regulation of phosphoinositol-3-kinase-related pathways [60]. Some particular MedDiet antioxidants such as flavonoids are able to directly modulate the disproportionate promotion of phosphoinositol-3-kinase-related pathways [61]. Second, monounsaturated (in olive oil) and polyunsaturated fatty acids (in nuts, fish, and seafood) contribute to reducing low-grade inflammation through their capacity to activate peroxisome-proliferator activated receptors [62] and bind fatty acid receptors in immune cells [63]. In addition, omega-3 polyunsaturated fatty acids can be transformed into anti-inflammatory eicosanoids such as 3-series prostaglandins and thromboxanes and 5-series leukotrienes [64]. Third, minor polar lipids present in several foods in the MedDiet (such as the glycerophospholipids, glycolipids, and betaine-related lipids in extra-virgin olive oil) are able to contribute to the anti-inflammatory potential of this dietary pattern due to their capacity to counteract the pro-inflammatory signaling pathways of platelet-activating factor [65,66]. Finally, short-chain fatty acids (such as butyric, propionic, and acetic acids, derived from the intestinal fermentation of dietary fiber by probiotic bacteria) and some phenolic compounds are able to induce the activation of AMP-activated protein kinase [67,68], a cellular metabolic regulator capable of counteracting an exaggerated inflammatory response through its ability to induce several anti-inflammatory responses [69].

Our study presents some limitations. First, WBC count alterations were not a predetermined endpoint in the PREDIMED trial and, therefore, these analyses should be considered as exploratory. Second, our cohort of participants, studied between 2003 and 2009, could possibly be outdated to argue the significance of our findings, and therefore our conclusions should be validated in a more recent population to confirm their robustness. Third, WBC counts fluctuate over time (due to infections, daily life activities, and the individuals’ mood) and WBC count alterations had a low incidence rate in our population. Therefore, our findings should be interpreted with caution. Fourth, WBC counts were measured in different recruiting centers, which could have increased data variability. However, all analyses were stratified by recruiting site and adjusted for baseline WBC count values, and the inter-center coefficient of variability for the mean count value obtained for each site throughout the whole study is low (3.70%). Fifth, generalizability of our results to other populations than middle-aged and older adults at high cardiovascular risk is limited. Sixth, we were only capable of reporting moderate effects on some of the study outcomes, considering that MedDiet interventions were based on modest real-life dietary modifications and that the control diet was already a healthy, low-fat dietary pattern. Finally, self-reported information on dietary adherence and leisure-time physical activity is subjected to bias and the risk of residual confounding.

## 5. Conclusions

Our findings indicate that following a MedDiet reduced the risk of developing leukopenia in middle-aged and older adults at high cardiovascular risk. Additionally, high MedDiet adherence was associated with a decreased incidence of any WBC count alteration and attenuated all-cause mortality risk linked to leukopenia. Our findings suggest that the MedDiet could be a recommended eating plan in middle-aged and older adults with cardiovascular comorbidities in which leukopenia may lead to serious health consequences.

## Figures and Tables

**Table 1:** Study population (*n* = 4192) by intervention group.

	All Participants (*n* = 4192)	MedDiet–EVOO ^1^ (*n* = 1439)	MedDiet–Nuts (*n* = 1328)	Control Diet (*n* = 1425)
Age (years), mean ± *SD*	67.1 ± 6.14	66.8 ± 6.08	66.9 ± 6.04	67.5 ± 6.28
Female sex, *n* (%)	2416 (57.6)	854 (59.3)	719 (54.1)	843 (59.2)
Diabetes, *n* (%)	1949 (46.5)	683 (47.5)	607 (45.7)	659 (46.2)
Hypercholesterolemia, *n* (%)	3103 (74.0)	1062 (73.8)	995 (74.9)	1046 (73.4)
Hypertriglyceridemia, *n* (%)	1269 (30.3)	437 (30.4)	402 (30.3)	430 (30.2)
Hypertension, *n* (%)	3512 (83.8)	1196 (83.1)	1114 (83.9)	1202 (84.4)
Smoking habit:				
Never smokers, *n* (%)	2580 (61.5)	892 (62.0)	796 (59.9)	892 (62.6)
Current smokers, *n* (%)	574 (13.7)	193 (13.4)	188 (14.2)	193 (13.5)
Former smokers, *n* (%)	1038 (24.8)	354 (24.6)	344 (25.9)	340 (23.9)
Weight status:				
Body mass index <25 kg/m^2^, *n* (%)	283 (6.75)	99 (6.88)	104 (7.83)	80 (5.61)
Overweight (25–29.9 kg/m^2^), *n* (%)	1857 (44.3)	642 (44.6)	604 (45.5)	611 (42.9)
Obesity (≥30 kg/m^2^), *n* (%)	2052 (49.0)	698 (48.5)	620 (46.7)	734 (51.5)
MedDiet adherence score, mean ± *SD*	8.62 ± 1.96	8.76 ± 1.99	8.70 ± 1.96	8.40 ± 1.91
Leisure-time physical activity (metabolic equivalents of task/minute/d), median (1st–3rd quartile)	168 (56.2–315)	176 (61.8–319)	183 (63.9–328)	150 (47.4–281)

^1^ MedDiet–EVOO: Mediterranean diet enriched with extra-virgin olive oil; MedDiet–Nuts: Mediterranean diet enriched with mixed nuts.

**Table 2:** Incidence of white blood cell count alterations in the study population^1^.

	Leukocytosis		Leukopenia		Severe Leukopenia	
	Cases/Total (Incidence Rate)	Hazard Ratio [95% CI]	Cases/Total (Incidence Rate)	Hazard Ratio [95% CI]	Cases/Total (Incidence Rate)	Hazard Ratio [95% CI]
Control diet	16/1034 (1.55%)	1 (Ref.)	48/948 (5.06%)	1 (Ref.)	13/1034 (1.26%)	1 (Ref.)
MedDiets combined	26/2156 (1.21%)	1.14 [0.59; 2.20]	65/1977 (3.29%)	0.54 [0.36; 0.80]	10/2156 (0.46%)	0.25 [0.10; 0.60]
MedDiet–EVOO	14/1154 (1.21%)	1.05 [0.48; 2.28]	41/1055 (3.89%)	0.59 [0.38; 0.93]	7/1154 (0.61%)	0.31 [0.11; 0.85]
MedDiet–Nuts	12/1002 (1.20%)	1.23 [0.56; 2.71]	24/922 (2.60%)	0.47 [0.29; 0.78]	3/1002 (0.30%)	0.18 [0.055; 0.60]

^1^ Hazard ratios were estimated by multivariable Cox proportional hazards regression models stratified by sex, recruitment site, and educational level, and adjusted for white blood cell count, age, diabetes, hypercholesterolemia, hypertriglyceridemia, hypertension, smoking habit, leisure-time physical activity, body mass index, hemoglobin levels, alcohol consumption (baseline values, all), and two propensity scores that used 30 baseline variables to estimate the probability of assignment to each of the intervention groups. We used robust standard errors to account for intra-cluster correlations. MedDiet–EVOO: Mediterranean diet enriched with extra-virgin olive oil; MedDiet–Nuts: Mediterranean diet enriched with mixed nuts.

## Data Availability

The dataset analyzed during the current study is not publicly available due to national data regulations and for ethical reasons, including that we do not have the explicit written consent of the study volunteers to make their deidentified data available at the end of the study. However, datasets and R codes of data management/transformation and statistical analyses can be requested by sending a letter to the PREDIMED Steering Committee (predimed-steering-committe@googlegroups.com). The request will then be passed to all the members of the committee for deliberation.

## Supplementary Materials

The following are available online at https://www.mdpi.com/article/10.3390/foods10061268/s1, Figure S1: Weighted Kaplan–Meier estimates of the cumulative incidence of leukocytosis (a), leukopenia (b), and severe leukopenia (c) in intervention groups; Table S1: Power analyses for all study determinations; Table S2: Comparison of study population with non-included PREDIMED participants; Table S3: Evolution over time of white blood cell count in the PREDIMED study groups; Table S4: Associations of white blood cell count alterations at baseline with the risk of all-cause mortality stratified by intervention group and cumulative adherence to the Mediterranean diet.
