# Supplementary material for: Mediterranean Diet and White Blood Cell Count—A Randomized Controlled Trial
Source: Foods. 2021 Jun 2;10(6):1268. doi: 10.3390/foods10061268 (PMC8227102; doi:10.3390/foods10061268)
Supplement: Supplementary file 1 [file foods-10-01268-s001.zip › SUPPLEMENTARY DATA.pdf]

SUPPLEMENTARY DATA

SUPPLEMENTARY TABLES

Supplementary Table S1. Power analyses for all study determinations

	Volunteers (control diet)	**MedDiets combined vs. control diet**				**MedDiet-EVOO vs. control diet**				**MedDiet-Nuts vs. control diet**			
		Volunteers (MedDiets combined)	Cases (control + MedDiets combined)	HR detectable with ≥80% power		Volunteers (MedDiet-EVOO)	Cases (control + MedDiet-EVOO)	HR detectable with ≥80% power		Volunteers (MedDiet-Nuts)	Cases (control + MedDiet-Nuts)	HR detectable with ≥80% power	
				<1	>1			<1	>1			<1	>1
Leukocytosis onset	1,034	2,156	42	≤0.43	≥3.42	1,154	30	≤0.33	≥3.20	1,002	28	≤0.30	≥3.19
Leukopenia onset	948	1,977	113	≤0.59	≥1.90	1,055	89	≤0.54	≥1.86	922	72	≤0.50	≥1.90
Severe leukopenia onset	1,034	2,156	23	≤0.32	≥8.67	1,154	20	≤0.24	≥4.65	1,002	16	≤0.17	≥5.43

MedDiet-EVOO: Mediterranean diet enriched with extra-virgin olive oil; *MedDiet-Nuts*: Mediterranean diet enriched with mixed nuts.

Supplementary Table S2. Comparison of study population with non-included PREDIMED participants.

	**Study population (*n*=4,192)**	**Non-included PREDIMED participants (*n*=3,255)**	***P*-value**
Age (years), mean ± SD	67.1 ± 6.14	66.9 ± 6.28	0.275
Female sex, *n* (%)	2416 (57.6)	1866 (57.3)	0.921
Diabetes, *n* (%)	1949 (46.5)	1683 (51.7)	<0.001
Hypercholesterolemia, *n* (%)	3103 (74.0)	2281 (70.1)	<0.001
Hypertriglyceridemia, *n* (%)	1269 (30.3)	866 (26.6)	<0.001
Hypertension, *n* (%)	3512 (83.8)	2651 (81.4)	0.006
Current smokers, *n* (%)	574 (13.7)	473 (14.5)	0.259
Obese individuals, *n* (%)	2052 (49.0)	1452 (44.6)	<0.001
MedDiet adherence score, mean ± SD	8.62 ± 1.96	8.75 ± 1.81	0.003
Leisure-time physical activity (metabolic equivalents of task·minute/d), median (1st-3rd quartile)	168 (56.2-315)	186 (72.7-328)	<0.001

MedDiet: Mediterranean diet; *MedDiet-EVOO*: Mediterranean diet intervention enriched with extra-virgin olive oil; *MedDiet-Nuts*: Mediterranean diet intervention enriched with mixed nuts.

Supplementary Table S3. Evolution over time of white blood cell count in the PREDIMED Study groups

	**Baseline (10^9 cells/L, mean ± SD)**	**1 year (10^9 cells/L, mean ± SD)**	**2-3 years (10^9 cells/L, mean ± SD)**	**4-6 years (10^9 cells/L, mean ± SD)**	**Time effect (10^9 cells/L·year, [95% CI])**	**Time*group effect (10^9 cells/L·year, [95% CI], vs. control diet)**
Control diet	6.69 ± 2.09	6.58 ± 1.81	6.60 ± 1.92	6.84 ± 2.17	+0.026 [0.003 to 0.049] (*P*-value= 0.023)	Ref.
MedDiets combined	6.55 ± 1.81	6.52 ± 1.84	6.51 ± 1.66	6.68 ± 1.89		0.012 [-0.015 to 0.039] (*P*-value= 0.380)
MedDiet-EVOO	6.51 ± 1.74	6.50 ± 1.83	6.51 ± 1.71	6.72 ± 2.09		0.023 [-0.007 to 0.053] (*P*-value= 0.124)
MedDiet-Nuts	6.58 ± 1.88	6.53 ± 1.86	6.51 ± 1.59	6.63 ± 1.61		-0.002 [-0.033 to 0.029] (*P*-value= 0.879)

Time effects and between-group changes were estimated by repeated measurement mixed models adjusted for white blood cell count at baseline, age, sex, recruitment site, educational level, diabetes, hypercholesterolemia, hypertriglyceridemia, hypertension, smoking habit, leisure-time physical activity, body mass index, hemoglobin levels, alcohol consumption (baseline values, all), and two propensity scores that used 30 baseline variables to estimate the probability of assignment to each of the intervention groups. *MedDiet-EVOO*: Mediterranean diet intervention enriched with virgin olive oil*; MedDiet-Nuts*: Mediterranean diet intervention enriched with mixed nuts.

Supplementary Table S4. Associations of white blood cell count alterations at baseline with the risk of all-cause mortality stratified by intervention group and cumulative adherence to the Mediterranean diet.

		Leukocytosis			Leukopenia		
Association of white blood cell count alterations (at baseline) with the incidence of major cardiovascular events							
	Presence of the alteration	Cases/Total (incidence rate)	HR [95% CI]	*P*-value	Cases/Total (incidence rate)	HR [95% CI]	*P*-value
	No	171/4,106 (4.16%)	1 (Ref.)	-	165/3,819 (4.32%)	1 (Ref.)	-
	Yes	8/86 (9.30%)	1.52 [0.68; 3.38]	0.308	14/373 (3.75%)	1.01 [0.57; 1.77]	0.978
Stratification in PREDIMED intervention groups							
	Presence of the alteration	Cases/Total (incidence rate)	HR [95% CI]	Interaction (*P*-value)	Cases/Total (incidence rate)	HR [95% CI]	Interaction (*P*-value)
Control diet	No	55/1,395 (3.94%)	1 (Ref.)		52/1,307 (3.98%)	1 (Ref.)	
	Yes	3/30 (10.0%)	1.44 [0.30; 6.89]		6/118 (5.08%)	1.40 [0.56; 3.50]	
MedDiet groups	No	116/2,711 (4.28%)	1 (Ref.)		113/2,512 (4.50%)	1 (Ref.)	
	Yes	5/56 (8.93%)	1.49 [0.55; 4.04]	0.985	8/255 (3.14%)	0.73 [0.34; 1.54]	0.281
Stratification in groups according to cumulative adherence to Mediterranean diet (below vs. above the median)							
	Presence of the alteration	Cases/Total (incidence rate)	HR [95% CI]	Interaction (*P*-value)	Cases/Total (incidence rate)	HR [95% CI]	Interaction (*P*-value)
Below the median	No	97/2,074 (4.68%)	1 (Ref.)		93/1,952 (4.76%)	1 (Ref.)	
	Yes	6/45 (13.3%)	1.58 [0.64; 3.91]		10/167 (5.99%)	1.80 [0.92; 3.55]	

Above the median	No	72/2,022 (3.56%)	1 (Ref.)		70/1,857 (3.77%)	1 (Ref.)	
	Yes	2/41 (4.88%)	1.01 [0.19; 5.42]	0.237	4/206 (1.94%)	0.54 [0.19; 1.54]	0.032

Hazard ratios were estimated by multivariable Cox proportional hazards regression models stratified by: sex, recruitment site, and educational level; and adjusted for: white blood cell counts, age, diabetes, hypercholesterolemia, hypertriglyceridemia, hypertension, smoking habit, leisure-time physical activity, body mass index, hemoglobin levels, and alcohol consumption (at baseline). Analyses stratified according to cumulative MedDiet adherence were further adjusted for PREDIMED intervention group. We used robust standard errors to account for intra-cluster correlations.

HR: hazard ratio; *MedDiet*: Mediterranean diet.

SUPPLEMENTARY FIGURES

Supplementary Figure S1. Weighted Kaplan-Meier estimates of the cumulative incidence of leukocytosis (A), leukopenia (B), and severe leukopenia (C) in intervention groups.

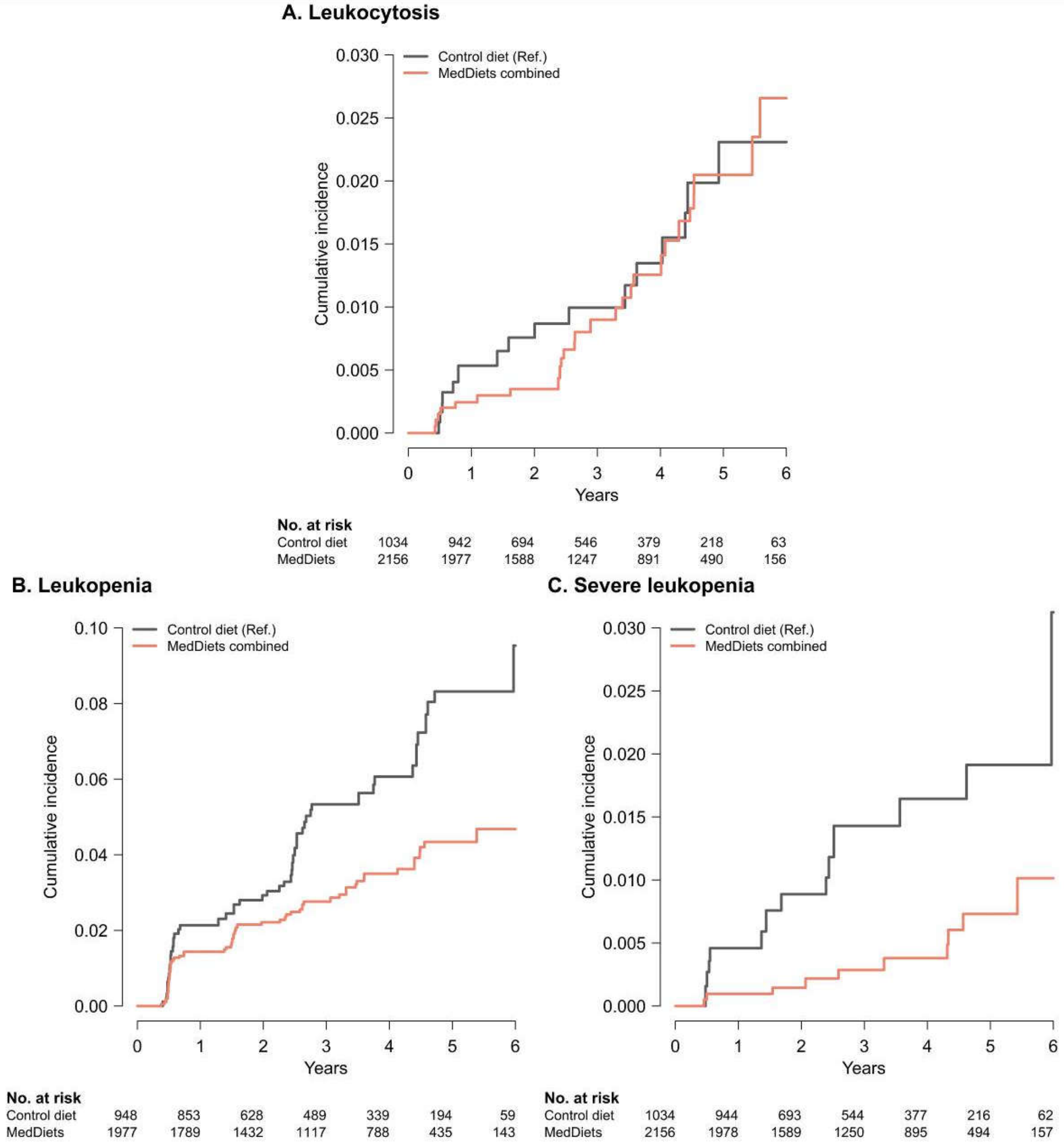

Kaplan-Meier curves weighted by inverse probability weighting using a propensity score model of assignment to intervention or control group based on: white blood cell counts at baseline, age, sex, recruitment site, educational level, diabetes, hypercholesterolemia, hypertriglyceridemia, hypertension, smoking habit, leisure-time physical activity, body mass index, hemoglobin levels, alcohol consumption (baseline values, all), and two propensity scores that used 30 baseline variables to estimate the probability of assignment to each of the intervention groups.
MedDiet-EVOO: Mediterranean diet enriched with extra-virgin olive oil; *MedDiet-Nuts*: Mediterranean diet enriched with mixed nuts.

Appendix. List of PREDIMED study collaborators

Hospital Clinic, Institut d'Investigacions Biomèdiques August Pi i Sunyer, Barcelona, Spain: R. Estruch, M. Serra, A. Pérez-Heras, C. Viñas, R. Casas, L. de Santamaría, S. Romero, E. Sacanella, G. Chiva, P. Valderas, S. Arranz, J.M. Baena, M. García, M. Oller, J. Amat, I. Duaso, Y. García, C. Iglesias, C. Simón, L. Quinzavos, L. Parra, M. Liroz, J. Benavent, J. Clos, I. Pla, M. Amorós, M.T. Bonet, M.T. Martin, M.S. Sánchez, J. Altirriba, E. Manzano, A. Altés, M. Cofán, C. Valls-Pedret, A. Sala-Vila, M. Doménech, R. Gilabert, and N. Bargalló.
University of Navarra, Primary Care Centres, Pamplona, Spain: M.Á. Martínez-González, A. Sánchez-Tainta, B. Sanjulián, E. Toledo, M. Bes-Rastrollo, A. Martí, C. Razquin, P. Buil-Cosiales, M. Serrano-Martínez, J. Díez-Espino, A. García-Arellano, I. Zazpe, F.J. Basterra-Gortari, E.H. Martínez-Lapiscina, A. Gea, M. Garcia-Lopez, J.M. Nuñez-Córdoba, N. Ortuño, N. Berrade, V. Extremera-Urabayen, C. Arroyo-Azpa, L García-Pérez, J. Villanueva-Tellería, F. Cortés-Ugalde, T. Sagredo-Arce, Mª D. García de la Noceda-Montoy, Mª D. Vigata-López, Mª T. Arceiz-Campo, A. Urtasun-Samper, Mª V. Gueto-Rubio, and B. Churio-Beraza.
University of Valencia, Valencia, Spain; Universitat Jaume I, Castellon, Spain; and Conselleria de Sanitat, Generalitat Valenciana: D. Corella, P. Carrasco, C. Ortega-Azorín, E.M. Asensio, R. Osma, R. Barragán, F. Francés, M. Guillén, J.I. González, C. Sáiz, O. Portolés, F.J. Giménez, O. Coltell (U. Jaume I), R. Fernández-Carrión, I. González-Monje, L. Quiles, V. Pascual, C. Riera, M.A. Pages, D. Godoy, A. Carratalá-Calvo, S. Sánchez-Navarro, and C. Valero-Barceló.
University Rovira i Virgili, Reus, Spain: J. Salas-Salvadó, M. Bulló, R. González, C. Molina, F. Márquez, N. Babio, M. Sorlí, J. García-Roselló, F. Martin, R. Tort, A. Isach, B. Costa, J.J. Cabré, J. Fernández-Ballart, N. Ibarrola, C. Alegret, P. Martínez, S. Millán, J.L. Piñol, J. Basora, and J.M. Hernández.
Institut Hospital del Mar d'Investigacions Mèdiques, Barcelona, Spain: M. Fitó, M.I. Covas, O. Castañer, S. Tello, J. Vila, H. Schröder, R. De la Torre, D. Muñoz-Aguayo, N. Molina, E. Maestre, A. Rovira, R. Elosua, and M. Farré.
University Hospital of Alava, Vitoria, Spain: F. Arós, I. Salaverría, T. del Hierro, J. Algorta, S. Francisco, A. Alonso-Gómez, J. San-Vicente, E. Sanz, I. Felipe, A. Alonso-Gómez, and A. Loma-Osorio.
University of Málaga, Málaga, Spain: E. Gómez-Gracia, R. Benítez-Pont, M. Bianchi-Alba, J. Fernández-Crehuet Navajas, J. Wärnberg, R. Gómez-Huelgas, J. Martínez-González, V. Velasco-García, J. de Diego-Salas, A. Baca-Osorio, J. Gil-Zarzosa, J.J. Sánchez-Luque, and E. Vargas-López.
Institute of Health Sciences, University of Balearic Islands, and Hospital Son Espases, Palma de Mallorca, Spain: M. Fiol, M. García-Valdueza, M. Moñino, A. Proenza, R. Prieto, G. Frontera, M. Ginard, F. Fiol, A. Jover, and J. García.
Department of Family Medicine, Primary Care Division of Sevilla, Sevilla, Spain: J. Lapetra, M. Leal, E. Martínez, J.M. Santos, M. Ortega-Calvo, P. Román, F.J. García, P. Iglesias, Y. Corchado, E. Mayoral, and C. Lama.

University of Las Palmas de Gran Canaria, Las Palmas, Spain: L. Serra-Majem, J. Álvarez-Pérez, E. Díez-Benítez, I. Bautista-Castaño, I. Maldonado-Díaz, A. Sánchez-Villegas, F. Sarmiendo-de la Fe, C. Simón-García, I. Falcón-Sanabria, B. Macías-Gutiérrez, and A.J. Santana-Santana.
Hospital Universitario de Bellvitge, Hospitalet de Llobregat, Barcelona, Spain: X. Pintó, E. de la Cruz, A. Galera, Y. Soler, F. Trias, I. Sarasa, E. Padres, R. Figueras, X. Solanich, R. Pujol and E. Corbella.
Clinical End Point Committee: F. Arós (chair), M. Aldamiz, A. Alonso-Gómez, J. Berjón, L. Forga, J. Gállego, M. A. García-Layana, A. Larrauri, J. Portu, J. Timiraus, and M. Serrano-Martínez.
